# Probing the digital exposome: associations of social media use patterns with youth mental health

**DOI:** 10.1038/s44277-024-00006-9

**Published:** 2024-04-23

**Authors:** David Pagliaccio, Kate T. Tran, Elina Visoki, Grace E. DiDomenico, Randy P. Auerbach, Ran Barzilay

**Affiliations:** 1 Division of Child and Adolescent Psychiatry, New York State Psychiatric Institute, New York, NY, USA; 2 Department of Psychiatry, Vagelos College of Physicians and Surgeons, Columbia University, New York, NY, USA; 3 Lifespan Brain Institute of the Children’s Hospital of Philadelphia and Penn Medicine, Philadelphia, PA, USA; 4 Children’s Hospital of Philadelphia, Department of Child and Adolescent Psychiatry and Behavioral Sciences, Philadelphia, PA, USA; 5 University of Pennsylvania Perelman School of Medicine, Department of Psychiatry, Philadelphia, PA, USA

## Abstract

Recently, the U.S. Surgeon General issued an advisory highlighting the lack of knowledge about the safety of ubiquitous social media use on adolescent mental health. For many youths, social media use can become excessive and can contribute to frequent exposure to adverse peer interactions (e.g., cyberbullying, and hate speech). Nonetheless, social media use is complex, and although there are clear challenges, it also can create critical new avenues for connection, particularly among marginalized youth. In the current project, we leverage a large nationally diverse sample of adolescents from the Adolescent Brain Cognitive Development (ABCD) Study assessed between 2019–2020 (*N* = 10,147, *M*_age_ = 12.0, 48% assigned female at birth, 20% Black, 20% Hispanic) to test the associations between specific facets of adolescent social media use (e.g., type of apps used, time spent, addictive patterns of use) and overall mental health. Specifically, a data-driven exposome-wide association was applied to generate digital exposomic risk scores that aggregate the cumulative burden of digital risk exposure. This included general usage, cyberbullying, having secret accounts, problematic/addictive use behavior, and other factors. In validation models, digital exposomic risk explained substantial variance in general child-reported psychopathology, and a history of suicide attempt, over and above sociodemographics, non-social screentime, and non-digital adversity (e.g., abuse, poverty). Furthermore, differences in digital exposomic scores also shed insight into mental health disparities, among youth of color and sexual and gender minority youth. Our work using a data-driven approach supports the notion that digital exposures, in particular social media use, contribute to the mental health burden of US adolescents.

## LAY SUMMARY

Smartphones and social media are increasingly central to teens’ social lives, leading to concerns about potential effects on mental health. Using a big-data approach, we created composite scores of digital exposures that related to poor mental health in a large national sample of youth. Use of certain apps, cyberbullying, having secret accounts and problematic/addictive social media use cumulatively related to worse outcomes, including the history of suicide attempts, beyond effects of non-social screentime and non-digital adversity.

## INTRODUCTION

The United States Surgeon General and leading pediatric health organizations have declared a national state of emergency regarding youth mental health [1–3], particularly raising concerns about the potential contributions of social media to mental health [4]. Spikes in depression and suicide rates have been observed in recent years, especially among youth of color and sexual and gender minority (SGM) adolescents [5–8]. Depression and other mental health concerns frequently onset during adolescence [9–11], which can be an especially stressful developmental period [12] as well as a critical time for identity and relationship formation [13–15]. Further research is needed to understand the potential contributions of social media on mental health among youth [16].

In recent decades, there have been major shifts in the centrality of digital devices to daily life and social relationships, particularly among adolescents. Over 95% of teens in the U.S. own smartphones [17, 18]. Smartphones have been increasingly available across income strata [17, 18] with nearly all adolescents reporting daily use, and a quarter reporting “almost constant” use [19]. Accordingly, concerns have been raised in the popular press about the potential negative effects of screentime (i.e., any activity on digital devices) on mental health and development [20, 21]. Screentime includes a wide ranges of activities, including passive video watching, texting, games, and social media, as well as educational and school-related activities. Data from the Adolescent Brain Cognitive Development (ABCD) Study shows annual increases in screen time across development (9–12-year-olds) [22].

Changes in our digital landscape have been particularly rapid regarding social media. Broadly, social media encapsulates digital platforms that help users develop social interaction or online presence [23]. This definition itself as well as teens’ preferred social media have evolved with the rapid shift from a small set of web-based platforms (e.g., MySpace) to the proliferation of smartphone-based apps (e.g., Instagram, TikTok). Despite increasing use, youth express an ambivalent need to devote time to social media to maintain peer relationships, while not enjoying using social media as much as other activities, e.g., listening to music [24]. Furthermore, significant sociodemographic differences have been observed;on average, boys tended to report increasing time on games and video watching whereas girls report increases in social activities (e.g., texting, and social media) [22]. White youth and those from high-income families typically have the greatest access to digital platforms, yet lower-income and youth of color often exhibit more screentime [22, 24]. Compared to their heterosexual peer, SGM teens report a greater likelihood of spending 3+ hours of non-school-related screentime daily (up to 85%) [5].

Given rising rates of mental health issues among adolescents [1, 3, 25, 26], concerns have been raised about the impact of digital technology. Despite widespread concerns, research has been inconclusive, yielding small or mixed-effects between social media and mental health [27–30]. Initial examination of ABCD data suggests only small associations between screentime and mental health [31–33]. Meta-analyses and large survey studies also suggest small associations between greater child and adolescent use of social media and worse depressive, internalizing, and externalizing symptoms, though substantial heterogeneity is noted [27, 29, 30, 34, 35]. This may be, in part, due to less time spent on in-person activities [36] or factors like social comparison [34, 37]. Longitudinal surveys provide mixed or null evidence on the directionality of these effects [35, 38–41]. Cross-sectional data from the 2021 CDC Youth Risk Behavior Survey show that serious consideration of attempting suicide was disproportionately reported among high schoolers reported 3+ hours/day of screentime, covarying race and sex (odds ratio [OR] = 1.68, *z* = 10.65, *p* < 0.001) [5]. However, it is not clear whether screentime, per se, is driving the association, or rather the association of screentime with suicidal risk is driven by specific types of use (e.g., adverse social media-related exposures).

**Fig. 1:**
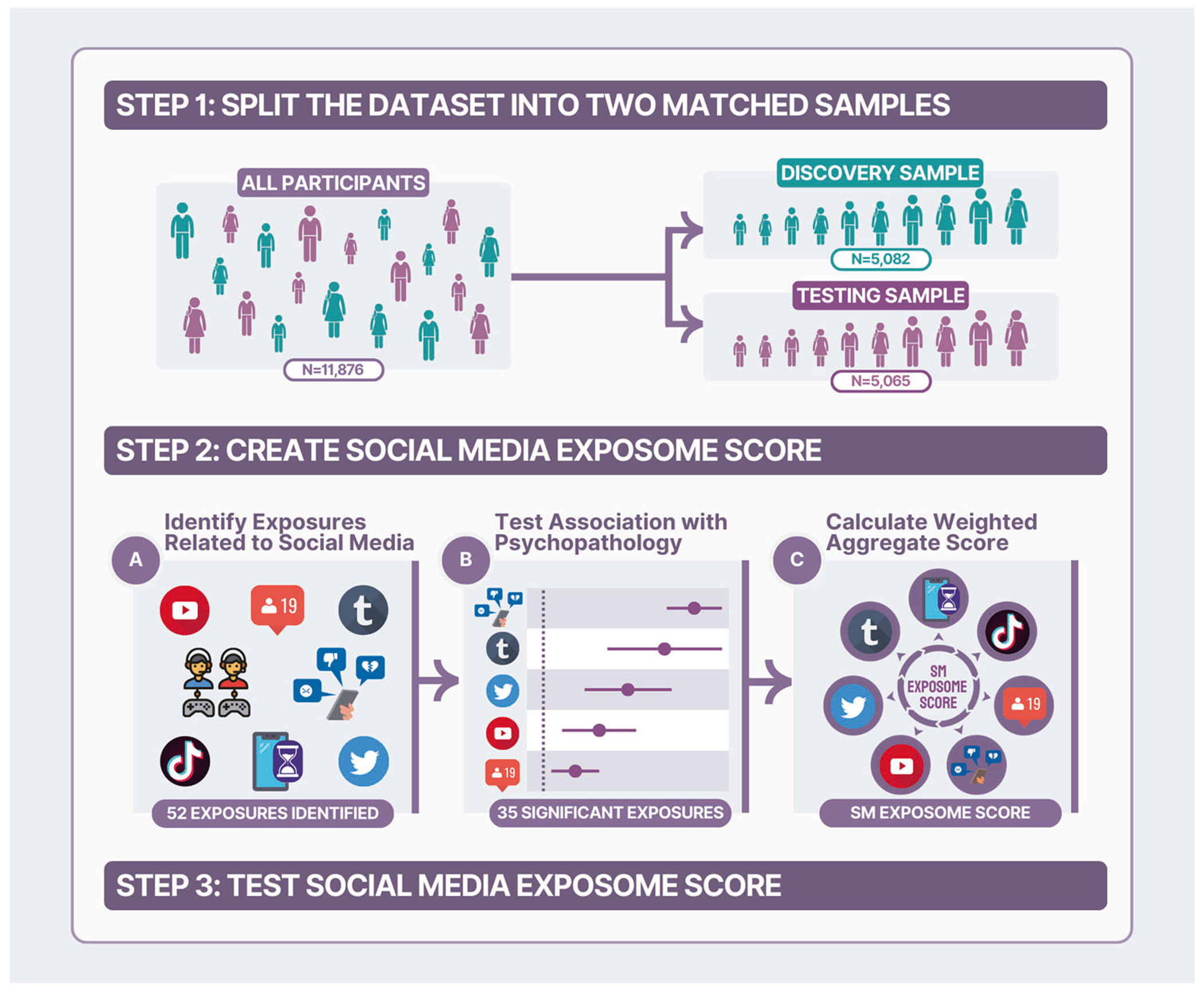
Conceptual overview. An overview of the flow of the analytic approach is presented here. The ABCD Study dataset was split into two independent subsamples for training and testing procedures (Step 1). We identified self- and parent-report variables that assessed digital and social media exposures (2A). Associations with mental health symptoms were assessed in separate models (2B). Weighted risk scores were aggregated from the significantly associated variables (2C). These aggregate risk scores were then validated and used in follow-up testing in the independent testing subsample.

Toward addressing this gap, we aimed to test the specificity of social media contributions to youth mental health, over and above general screentime, and non-digital adversity (e.g., abuse, trauma, neighborhood poverty, discrimination) [42]. We leveraged ABCD data that includes a large sample of diverse youth from across the U.S. We utilized data-driven exposome-wide association study (ExWAS) analyses to test cross-sectional associations of multiple measures of social media use with mental health at age 12. Previous ExWAS have examined environmental and lifestyle factors to explain variance in physical health conditions [43, 44] and, more recently, mental health [45, 46]. This approach can help advance the field which mostly focuses on single digital exposures in isolation (e.g., cyberbullying, addictive social media use) and can address some challenges of single-exposure studies [47–49], including multiple comparisons and collinearity. We used ExWAS findings to construct dimensional digital exposomic risk scores that aggregate an individual’s associated mental health risk and apply them for more parsimonious follow-up tests (Fig. 1). Specifically, we hypothesized that specific aspects of social media exposure would specifically relate to worse mental health, more so than general screentime, and separable from effects of non-digital adversity [42]. Furthermore, given known disparities in mental health outcomes by gender, race, and SGM identity [5–9, 50–53], we hypothesized differential exposure [54] based on the exposomic risk scores across these subpopulations (e.g., greater social media exposure among girls than boys) as well as potential differential effects whereby the association between digital exposures and mental health varies by identity (e.g., stronger links between exposure and poor mental health among SGM than non-SGM youth). These analyses will lay the groundwork for future longitudinal and causal analyses.

## METHODS

### Participants

We included ABCD Study participants who completed the 2-year follow-up that included assessment of screentime and social media use (*N* = 10,147) [55–57]. Data were collected between 2019–2020 and drawn from the ABCD Study’s Data Release 4.0 (https://doi.org/10.15154/1523041). Briefly, the ABCD Study is a collaborative project with the goals of understanding: (a) normal variability and (b) environmental and socioemotional factors that influence brain and cognitive development [58]. Starting in 2016, ABCD recruited diverse children (*N* = 11,876) ages 9–10 through schools near 21 sites across the US [59].

#### Clinical outcomes.

Our primary outcome was self-reported youth psychopathology assessed through the Brief Problems Monitor (BPM) [60]. This measure assesses general functioning and mental health, including items refined from the Child Behavior Checklist (CBCL) [61]. Specifically, we focused on age-and sex-normed Total Problems *T*-scores, which reflect a combination of internalizing, externalizing, and attentional problems. In sensitivity analyses, we examined BPM Internalizing *T*-scores and parent-report CBCL Total Problems *T*-scores [42]. Follow-up analyses examined self-reported suicide attempts as a higher severity outcome, based on the computerized Kiddie-Structured Assessment for Affective Disorders and Schizophrenia for DSM-5 (KSADS-5) [62, 63].

#### Digital exposures.

ABCD collects youth- and parent-report data on digital experiences, including the Cyber Bullying Questionnaire [64] and Youth and Parent Screen Time Surveys [22]. Individual variables were refined for analysis by the co-authors. For example, redundant or branching items and variables with <1% endorsement were removed. Screentime assessments included separate hour and minute response options, which were combined for analysis. Extreme outliers on continuous response variables were removed (e.g., number of social media followers; see Supplement).

We identified 52 digital and social media exposure variables; after cleaning, 41 variables were retained for analysis (Table S1), which captured five broad domains: (1) screentime (i.e., minutes/hours per day by weekend/weekday), (2) parental monitoring (e.g., “*Do you suspect that your child has social media accounts that you are unaware of?*”), (3) apps used (e.g., yes/no has an Instagram account), (4) overuse/addictive patterns of use (e.g., “*I use social media apps so much that it has had a bad effect on my schoolwork or job*”), and (5) peer interaction (e.g., “*I feel connected to others when I am using my phone*”). Total screentime for non-social purposes (e.g., for schoolwork) was extracted as a covariate using 13 items from the Youth/Parent Screen Time Surveys (Supplement).

### Statistical analysis

#### ExWAS.

All analyses were conducted in R [65]. Building on prior ExWAS research [42, 43, 66–68], our analytic plan applied the following steps (Fig. 1): (a) the ABCD dataset was split into training (*n* = 5082) and testing (*n* = 5065) subsamples using the ABCD Reproducible Matched Samples (ABCD_3165 collection [69]), matched across study sites on age, sex, ethnicity, grade, parent education, family income, and family-relatedness; (b) missing digital exposure data was non-parametrically imputed for both subsamples separately (*missForest::RandomForest* [70]; mental health outcome variables were not imputed); (c) collinear (Pearson’s *r* > 0.9) exposures in the training sample were removed (*caret::findCorrelation* [71], as in prior work [46]), (d) each digital exposure was tested as an independent variable in a separate linear-mixed-effects model (LME; *lme4::lmer*; [72]) with BPM-*T*-scores as the dependent variable in the training sample, with random intercepts for family nested within study site and covariates for age, sex, race (binary variables for identifying as Black and as White), and Hispanic ethnicity, (e) FDR-correction for 41 comparisons was applied to identify significant exposures as risk (coefficient>0) or protective (coefficient<0) factors, and (f) aggregate digital exposomic risk scores were derived for each participant in the testing subsample as the sum of each variable multiplied its coefficient from ExWAS LME models; higher scores indicate greater digital exposomic risk for mental health problems.

#### Validation.

In the independent testing sample, successive LME models were used to validate the specific association between aggregate digital exposomic risk scores and BPM Total Problems *T*-scores, over and above demographics, non-social screentime, and childhood non-digital adversity, calculated in our previous work [42]. All models included random effects for families nested within the study site as well as fixed-effect covariates for age, sex, race, Hispanic ethnicity, annual household income (ordinal variable, from below $5,000 (1) to above $200,000 (10)), and parent education (data at 1-year assessment, mean of the highest grade or degree that parent(s) completed). We first estimated a model that included demographics (Model-1), then added total non-social screentime (Model-2), and then added a measure of childhood adversity that aggregates environmental burden captured by age 11 [42, 66] (Model-3). Last, digital exposomic risk scores were entered to examine the added variance explained by mental health burden (Model-4). Nakagawa marginal R^2^ indicated the variance explained by the fixed effects [73]. All model coefficients and odds ratios are presented with their 95% confidence interval (CI) and adjusted for covariates.

#### Suicide attempts analyses.

To address the potential contribution of digital exposomic risk to suicide attempts, we estimated logistic regression models with self-reported lifetime suicide attempt history (K-SADS) as the dependent variable (instead of BPM-*T*-score as above).

#### Disparities in digital exposomic risk across subpopulations.

To examine differential exposure, we first compared digital exposomic risk scores across populations in the testing subsample, based on sex, race/ethnicity (groupings for non-Hispanic White, non-Hispanic Black, and Hispanic), and SGM identity [74]. Non-parametric tests were used with their corresponding effect sizes, specifically Kruskal-Wallis (*χ*^2^) across three race/ethnicity groups and Dunn’s Kruskal–Wallis Multiple Comparisons test with Holm-adjusted *p*-values for pairwise comparisons across race/ethnicity groups, and Mann–Whitney tests for two-group comparisons (Glass rank biserial coefficient $\hat{r}$). To examine the differential effects of digital exposomic risk across subpopulations, we added interaction terms to the main LME models between exposomic risk scores and sex, race/ethnicity, and SGM identity. Significant interactions suggest that the association between digital exposomic risk and mental health differs across populations. We further parsed significant interactions in stratified subgroup analyses.

#### Sensitivity analyses.

First, we re-examined our main validation model without the removal of outlier values. Similarly, we re-ran our main ExWAS with list-wise deletion rather than multiple imputations. To address possible biases based on outcome measure selection, we re-examined the main validation analyses using self-reports of internalizing symptoms and parent-reported CBCL Total Problems *T*-scores as outcomes. Furthermore, given the variable nature of digital exposures in this age group, we also examined sensitivity analyses in subsamples of children excluding those (a) who do not have a personal smartphone and (b) do not use social media (see Supplement). Finally, to address unmeasured confounding, we conducted an *E*-value analysis [75] on our main Model-4. The *E*-value approach probes confounding of binary outcomes on a risk-ratio scaling; thus, we conducted a logistic regression (with all covariates in Model-4) with BPM Total Problems *T*-scores as a binarized outcome comparing the top 10% as high scores against the remaining 90% as the reference.

## RESULTS

### Participants

A summary of demographics, clinical scores, and general screentime is presented in Table 1, split into training and testing samples. No significant differences were noted between subsamples (all ps > 0.05).

### ExWAS (training sample)

**Fig. 2:**
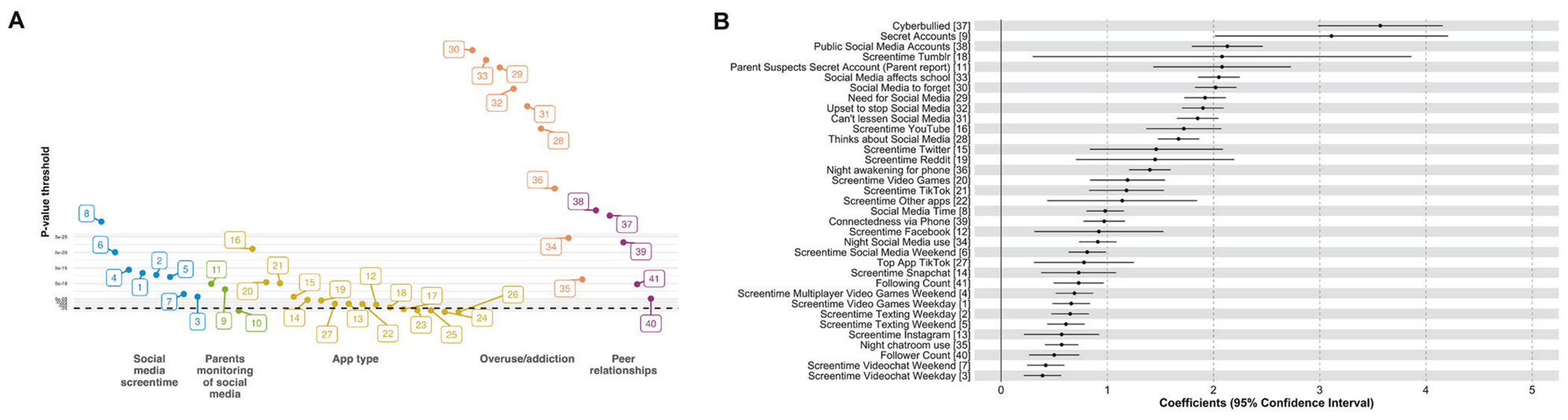
Digital ExWAS Results. Results of ExWAS analysis in the testing subsample are displayed here summarizing variables that exhibited an FDR-corrected significant association with Brief Problems Monitor (BPM) total *T*-scores. Panel **A** displays the significance of these associations in a Manhattan plot with *p*-values from individual linear-mixed-effects models on the *y*-axis with a log-transformed scale. Variables are arranged into conceptual categories. Panel **B** shows the magnitude of these associations in a forest plot with the linear-mixed-effects model coefficient and associated 95% confidence interval. Zero indicates no association between exposure and mental health. All variables identified exhibited a positive association such that higher values (or ‘yes’ endorsement) were associated with greater mental health burden. Variables are numbered to match the listing in Table S1.

Of the 52 digital and social media exposure variables examined, 7 were removed for low endorsement (<1%), and 4 were removed given high collinearity (*r* > |0.9 |; Fig. S1). Of the 41 remaining variables included in the ExWAS, 35 showed FDR-corrected-*p* < 0.05 significant associations with overall mental health in separate models, measured using the self-reported BPM Total Problem *T*-scores, (Fig. 2 and Table S1). Highly significant risk-related exposures included weekday videogame screentime, having social media accounts secret from one’s parents, addictive social media use, and experiencing cyberbullying (i.e., all showed associations between greater exposure and higher Total Problem scores;Fig. 2b). Experiencing cyberbullying and having social media accounts secret from one’s parents showed the highest association with worse BPM-*T*-scores. Having a private (i.e., viewable by friends only) vs. public social media account exhibited a protective association with BPM-*T*-scores.

### Digital exposomic risk score validation (testing sample)

Following the ExWAS, we calculated an aggregate digital exposomic risk score per participant. To validate the exposomic risk scores in the independent testing sample and determine their specificity, we estimated 4 LME models and examined the variance explained by mental health burden (Table S2; Fig. S2). Demographic variables accounted for minimal variance in BPM-*T*-scores (1.14%; Model-1). Non-social total screentime was significantly associated with higher BPM-*T*-scores (*b* = 1.38, 95% CI = [1.19–1.56], *t* = 14.46, *p* < 0.001, Model-2), and significantly increased variance explained in BPM-*T*-score to 6.18%. Non-digital childhood adversity was also significantly associated with higher BPM-*T*-scores (*b* = 1.31, 95%CI = [1.07–1.56], *t* = 10.65, *p* < 0.001, Model-3) and significantly increased the variance explained in BPM-*T*-score to 9.07%. Critically, digital exposomic risk scores are significantly associated with higher BPM-*T*-scores (estimate = 1.78, 95%CI = [1.58–1.98], *t* = 17.41, *p* < 0.001, Model-4; Table 2), over and above these other factors, and significantly increased variance explained in mental health burden to 15.61% (Fig. S2).

### Association of digital exposomic risk with suicide attempts

We tested the association of digital exposomic risk scores with youth-reported lifetime suicide attempts (Table S3). Higher digital exposomic scores are significantly associated with higher odds of reporting a prior suicide attempt (OR = 1.76, 95%CI = [1.39–2.23], *z* = 4.69, *p* < 0.001), while covarying demographics and non-social screentime (OR = 1.10, CI = [0.82–1.47], *z* = 0.63, *p* = 0.53, Table S3). This association further remained significant when covarying for non-digital childhood adversity (OR = 2.76, CI = [1.88–4.05], *z* = 5.18, *p* < 0.001; Table S3), which did strongly relate to suicide attempt history.

### Disparities in digital exposomic risk

**Fig. 3:**
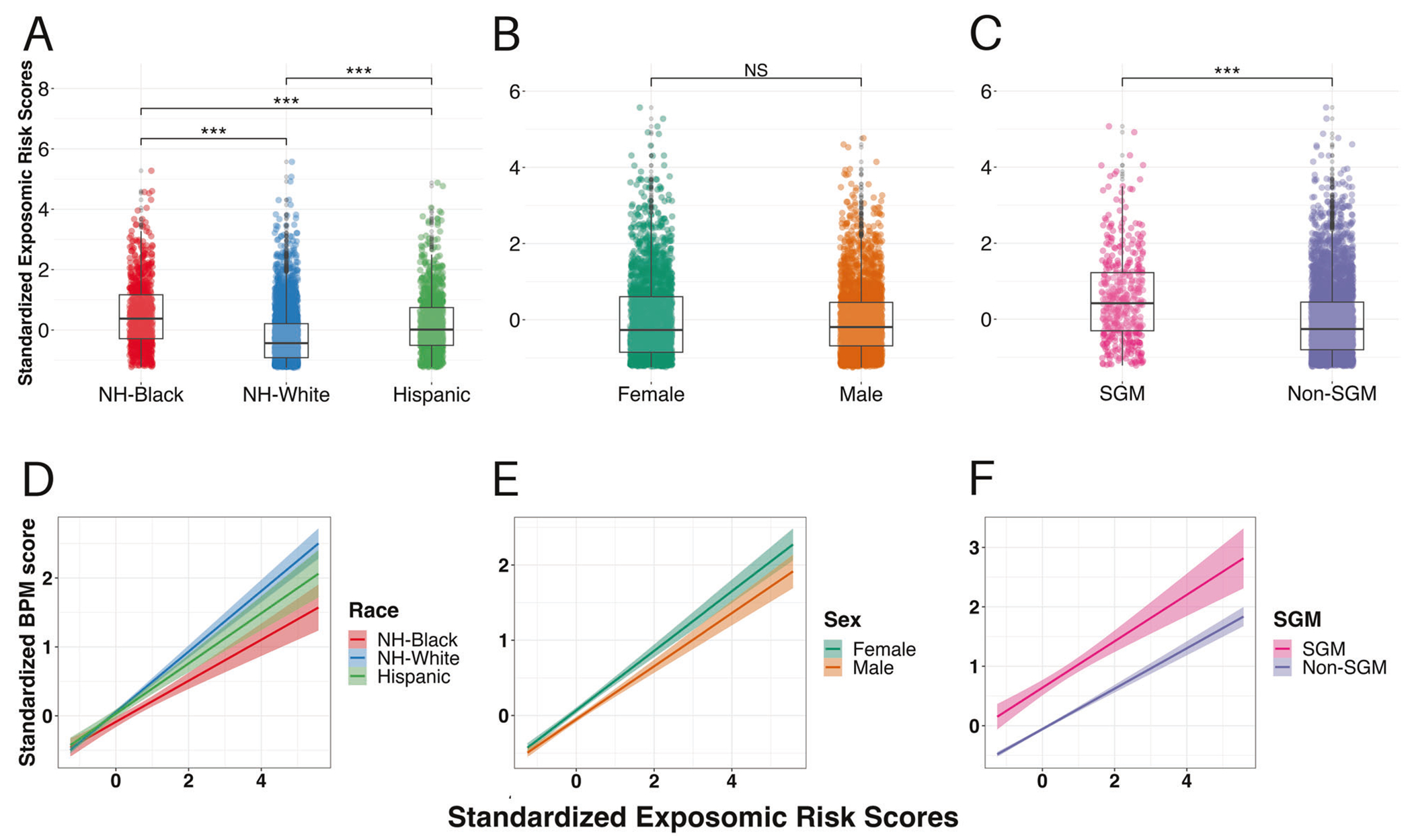
Differential exposure and effects by race, sex, and SGM identity. The top row of figures displays exposomic risk scores in the testing subsample split by **A** race/ethnicity, **B** sex, and **C** sexual and gender minority (SGM) identity. There was significant group difference based on race/ethnicity (non-Hispanic [NH]-Black > Hispanic > NH-White; Kruskal–Wallis *p* < 0.001, post hoc pairwise Dunn’s Tests for Multiple Comparisons Holm-adjusted-*p* < 0.001 for all comparisons). No sex differences were observed (Mann–Whitney *p* = 0.17). SGM youth had greater digital exposomic scores compared to their peers (Mann–Whitney *p* < 0.001). Points represent individual youth’s scores along with box-and-whisker plots. The bottom row of figures displays the simple slope (and 95% confidence interval in the shaded region) of the association between digital exposomic risk scores and Brief Problems Monitor (BPM) total *T*-scores based on **D** race/ethnicity, **E** sex, and **F** SGM identity. Black youth showed a weaker association between digital exposomic risk and BPM scores (digital exposome by Black race interaction *p* < 0.001). No significant differential associations were observed based on ethnicity, sex, or SGM identity. ***p < 0.001.

Examination of differential exposure to social media exposomic risk in the testing sample revealed that the digital exposomic risk scores were highest among youth identifying as Non-Hispanic Black (median = 0.38), compared to Non-Hispanic White (median = −0.44) and Hispanic youth (median = 0.01), with Hispanic youth having greater scores than Non-Hispanic White youth ($\chi_{\text{Kruskal–Wallis}}^{2}(2)=480.90$, *p* < 0.001; all pairwise Holm-adjusted-*p* < 0.001; Fig. 3A). There were no sex differences in exposomic risk scores (median female = −0.27, male = −0.19, *W*_Mann-Whitney_ = 3,124,298, $\hat{r}=-0.022$, *p* = 0.17; Fig. 3B). Youth identifying as SGM had significantly greater exposomic risk scores compared to their peers (median SGM = 0.42, non-SGM = −0.26, *W*_Mann-Whitney_ = 1,223,454, $\hat{r}=-0.33$, *p* < 0.001; Fig. 3C).

Examination of differential effects of the association between the digital exposomic risk scores and mental health burden revealed a significant digital exposome-by-Black race interaction (estimate = −0.12, *t* = −3.37, *p* = 0.001; Fig. 3D), such that Black youth exhibited weaker association between digital exposomic risk scores and BPM score, with no significant differential associations among Hispanic youth (digital exposome-by-Hispanic ethnicity interaction, *p* = 0.47; Fig. 3D). There was no sex difference in the association between the digital exposomic scores and BPM-*T*-scores (Fig. 3E, exposure-by-sex interaction; estimate = −0.03, *t* = −1.13, *p* = 0.26) and no differential associations among youth identifying as SGM (Fig. 3F, exposure-by-SGM interaction; estimate = −0.02, *t* = −0.46, *p* = 0.65).

### Sensitivity analyses

Digital exposomic risk scores remained significantly associated with mental health burdens in multiple sensitivity analyses. Specifically, main analysis Model-4 remained the same when not removing potential outliers from the dataset (Table S4) and when excluding children who did not report having a smartphone or those who do not use social media (Tables S5 and S6). Main analyses were confirmed when using list-wise deletion rather than multiple imputations. This sensitivity analysis highlighted 31 variables passing multiple comparisons correction (compared to 35 in the main analysis) in the ExWAS in the training subsample (Table S1). Exposomic risk scores were similarly related to BPM total problem *T*-scores when not imputing the testing subsample (estimate=1.44, 95%CI = [1.25-1.62], *t* = 15.09, *p* < 0.001). Furthermore, digital exposomic risk scores were significantly associated with both self-reported BPM Internalizing *T*-scores (Table S7) and parent-reported CBCL total problem *T*-scores, though accounting for less variance than in BPM total scores (Table S8). Finally, in *E*-value analyses, higher digital exposomic risk scores related to higher likelihood of exhibiting high BPM-*T*-scores (i.e., in the top 10% of scores; OR = 1.95; 95%CI = [1.73–2.20]), covarying for demographics, non-social screen time, and childhood adversity. An unmeasured confounder would have to be associated with 3.3-fold (lower limit of 95%CI = 2.9) increases in both exposome risk scores and likelihood of exhibiting high BPM scores to explain away the observed effect, above and beyond the measured confounders.

## DISCUSSION

Current findings highlight associations between digital exposures and mental health in a large national sample of youth. Furthermore, associations remained significant beyond the effects of general screentime and non-digital childhood adversity, suggesting that the digital exposome adds a specific component to the mental health burden of American youth, consistent with concerns raised by the U.S. Surgeon General [4]. Social media and other digital exposures are often inter-related and exist within rapidly changing digital landscapes, necessitating analytic methods that do not focus on specific exposures in isolation. Thus, the ExWAS addresses this challenge with data-driven approaches to identify and weigh relative associations of various digital behaviors with mental health. The current results highlight the utility of the ExWAS to develop aggregate risk scores that explained significant variance in mental health burden in independent subsamples of youth. Notably, digital exposomic risk scores are also associated with increased odds of suicide attempts, in contrast to non-social screentime. This suggests that it is not screentime per se that contributes to risk, but rather that the type of digital behavior is critical to consider. We used digital exposomic risk scores further to illuminate disparities in exposure and associations with mental health across sex, race, and SGM identity. Our findings add key insights regarding the association between digital exposures and early adolescent mental health, which is a critical pediatric health problem [4, 16]. Taken together, this work can help to develop richer theoretical models to guide the development of prevention and intervention programs.

The ABCD Study provides access to a large, national dataset examining a critical period of development. This allows for a powerful analysis of associations between digital exposures and mental health across the U.S. with an appropriate sample size to pursue independent model testing and validation. First, we began by screening available measures of digital exposures from child- and parent-report in relation to overall mental health severity. Data-driven ExWAS analyses identified a combination of common exposures of smaller effects and rarer exposures of larger effects. For example, 48% of youth in ABCD reported having a public (vs. private) account on their most frequently used social media platform, which related to negative mental health outcomes at a smaller effect size (estimate = 2.13 *T*-score points higher on the BPM on average). On the other hand, 9% of youth report being the target of cyberbullying, which is associated with larger differences in negative mental health outcomes (estimate = 3.57). This bolsters confidence in the validity of the ExWAS approach as cyberbullying is an established risk factor for depression and suicide in youth [76–79].

Examination of individual exposures identified in the ExWAS revealed that different facets of social media contribute to mental health. First, as expected, the subjective feeling that one’s social media use is becoming compulsive and interferes with daily activities (e.g., schoolwork) is related to worse mental health. Endorsement of these feelings and behaviors indicates a clear need for intervention to mitigate problematic use and its underlying causes. Excessive use may include nighttime use, which can impact sleep with potential consequences for mental health [80], with known implications for suicidal behaviors [81–83]. Second, parental monitoring of youth social media is also associated with mental health. About 7% of parents suspected that their child had social media account(s) that they were not aware of, with 15% of youth reporting having secret accounts. Both factors are associated with greater youth psychopathology. Although current data did not allow insight into motivations behind keeping secret accounts (e.g., breaking parental rules, accessing mature content), this underscores the importance of developing parent training guidelines to support healthy adolescent social media use.

Our findings begin to provide insights into the association of specific apps with youth mental health, but this must be contextualized within large inter-individual differences and trends over time. In the current sample,16% of youth-reported TikTok as their most used social media app. TikTok became available for download internationally in 2017 and rose to popularity in the U.S. after merging with musical.ly in 2018. Thus, the current data represent a snapshot of TikTok usage during a highly transitional time and its association with youth mental health will need to be monitored in later ABCD data and other future studies. Furthermore, TikTok, Instagram, and Snapchat usage have largely supplanted other platforms for youth [17]. Few youths ( < 1%) reported Facebook as their most used social media, and thus, this variable was pruned from analyses. It will be important to distinguish types of usage in future work [84–86], e.g., effects of TikTok and YouTube may be particularly driven by passive scrolling and watching behaviors (vs. more active use or socialization). We did observe a strong but variable association between Tumblr screentime and mental health. This, again, may reflect inter-individual differences and changing trends in usage. Beginning in 2018, Tumblr faced drops in userbase following major changes in content moderation, corporate ownership, and pushback on changes from SGM communities.

Our aggregate weighted digital exposomic risk scores facilitated comprehensive testing of sociodemographic disparities [5–8, 51, 52]. This approach can be preferable to analyzing individual, correlated exposures in isolation (due to smaller sample sizes and multiple testing). Analyses examined differences in the magnitude of exposure (differential exposure) and associations with mental health (differential effects) across race/ethnicity, sex, and SGM identity. We did not observe differences between males and females in exposure scores nor the association between social media and mental health. Given known sex differences in mental health [9, 50, 53], future work should continue to probe how social media could contribute, particularly across the pubertal transition [87]. Nonetheless, Black youth and youth who identify as SGM exhibited greater digital exposomic risk scores compared to their peers. Yet, interestingly, Black youth may exhibit weaker associations between social media exposure and mental health. This may be due to various factors, including access to supportive content and communities via social media, greater salience of non-digital risk factors, etc. Clinically, given the crisis around mental health and suicide among Black youth [88, 89], our findings may nonetheless suggest that clinicians should be aware of digital risk exposure in these populations. Additionally, whereas SGM youth experienced a greater burden of digital exposomic risk, they did not display differential associations between digital exposomic scores and mental health than non-SGM youth, thus higher differential exposure may contribute to higher mental health burden among SGM youth (rather than differential mechanisms), which does align with some conceptual models of SGM mental health [90]. Our findings add to previous ABCD analyses showing that SGM youth also report more offline adverse experiences [74]. Note that the ABCD assessments do not specifically ascertain exposure to SGM minority stress [52, 74, 90, 91] that may be disproportionally experienced even in digital environments. Additionally, the greater digital exposome burden of SGM youth calls for a deeper examination of the online experiences of LGBTQ + youth and how this may affect mental health, particularly during identity formation and coming out. In terms of public health, our results call for more research on the causal role of digital exposures in youth mental health and suicide risk, as mitigating exposure to digital stressors is a potentially modifiable risk factor for minoritized groups.

There are several limitations, which may guide future studies. First, the current analyses leveraged cross-sectional ABCD data. Additional social media assessments should be examined from later waves of ABCD in future longitudinal work. Particularly, longitudinal models can help examine the directionality of associations, potential causal effects, and changes in associations over age and puberty. Second, although we removed highly colinear exposure variables, the ExWAS-derived scores do not fully account for collinearity among exposures. The current scores remain interpretable in their construction, but other approaches to modeling the exposome accounting for its correlated structure [42, 92] can be examined in the future. Last, ABCD was not designed specifically to interrogate social media and digital exposures, so assessments were limited in scope and depth. Though a diverse range of behaviors were examined, our results highlight areas that can be probed more deeply in future work. Similarly, the available measures relied on self- and parent-report, which can be supplemented by other types of digital phenotyping in the future [93–95]. Nonetheless, our sensitivity analyses do highlight that exposomic risk is also related to parent-reported psychopathology mitigating potential concerns about shared method variance between adolescent self-reports of exposure and BPM. It will be important to confirm the current results in independent samples to test the robustness of the findings as well as to examine generalizability to other populations that may differ in their access to and relationships with digital exposures.

Additionally, future work should examine exposures that reflect positive or resilience-promoting activities. Critically, social media creates new avenues for youth to establish and maintain social networks [19], which often do not differ in quality from offline peer relationships [96]. This became increasingly salient during COVID-19 pandemic lockdowns as digital communication became a positive force and lifeline for many people [97, 98]. Furthermore, social media can be highly beneficial to facilitating community building and advocacy work, allowing Black youth to connect across geographic boundaries [99, 100]. Similarly, SGM youth can especially benefit from online platforms, including by viewing informative/educational content supporting their identity formation, finding peer support or role models, and navigating the coming out process [101–103]. Interestingly, prior work does suggest that increased screentime does not displace other types of recreational activities [104], yet social media remains important for building in-person relationships.

In summary, this work provides the first ExWAS approach to understanding risk factors strictly from the “digital world” in this large national dataset and offers potential inroads for developing public health strategies to support adolescent mental health. This is in line with growing recent concerns about the potential negative effects of social media and online content on mental health, as highlighted by the American Psychological Association for the U.S. Senate Judiciary Committee [105]. To address these concerns, we must pursue granular parsing of screentime and related behaviors to identify specific and modifiable mechanisms. This must be continually updated and contextualized within rapidly changing digital landscapes. Digital technology will continue to be central to social relationships, and we also cannot discount the potential benefits of digital technologies and social media. Separating nuances in use patterns may be critical, including active vs. passive usage [85, 86], public vs. private accounts, and weekday vs. weekend patterns. Understanding the reasons for social media use can also be important, as seeking social connection may relate to problematic outcomes [106]. As not all platforms are equivalent and a given platform can facilitate a wide range of adolescent behaviors, future work should aim to take dynamic and idiographic approaches grounded in current adolescent lived experiences, and can also apply multi-modal approaches leveraging smartphone sensors or wearables [107, 108] to gain comprehensive picture of digital exposures in youth.

## Supplementary Material

Supplementary Materials

## Figures and Tables

**Table 1.** Sample characteristics by ABCD study subsamples.

	Training (*n* = 5082)	Testing (*n* = 5065)	*p*-value
Age (*M* (SD) years)	12.01 (0.66)	12.00 (0.66)	0.332
Sex (*N* (%) female)	2446 (48.13%)	2385 (47.09%)	0.302
SGM identity (*N* (%))	406 (7.99%)	406 (8.02%)	0.998
Race-White (*N* (%))	3901 (76.76%)	3836 (75.74%)	0.236
Race-Black (*N* (%))	985 (19.38%)	1014 (20.02%)	0.437
Race-American Indian/Alaska Native (*N* (%))	169 (3.33%)	191 (3.77%)	0.247
Race-Native Hawaiian or Pacific Islander (*N* (%))	34 (0.67%)	28 (0.55%)	0.532
Race-Asian (*N* (%))	313 (6.16%)	321 (6.34%)	0.743
Race-other (*N* (%))	317 (6.24%)	325 (6.42%)	0.744
Race-mixed (*N* (%))	608 (11.96%)	626 (12.36%)	0.565
Hispanic ethnicity (*N* (%))	1014 (19.95%)	1018 (20.10%)	0.929
Non-social screentime hours/week (*M* (SD))	24.88 (13.97)	25.40 (14.46)	0.069
Childhood adversity exposome (*M* (SD))	−0.03 (0.99)	−0.03 (0.99)	0.902

*Note*. Characteristics of the sample are displayed split by the training and testing subsamples. No significant differences were noted between these subsamples. Means (*M*) and standard deviations (SD) are presented for continuous variables; *p*-values represent *t*-tests of group differences in continuous variables. Count (*N*) and percent (%) are presented for binary variables; *p*-values represent chi-squared tests of group differences in binary variables.

**Table 2.** Association of the digital exposomic score with overall psychopathology (BPM-*T*-score) in the independent testing ABCD subsample.

	Estimate	95% CI	*t*-stat	*p*-value
Intercept	50.52	47.25–53.79	30.28	**<0.001**
Age (years)	0.00	−0.02 to 0.02	−0.17	0.868
Sex (male > female)	0.30	−0.03 to 0.62	1.81	0.071
Race-Black	−1.46	−2.05 to −0.87	−4.84	**<0.001**
Race-White	0.39	−0.14 to 0.91	1.45	0.147
Hispanic ethnicity	0.20	−0.28 to 0.68	0.82	0.414
Household income	0.15	0.04–0.25	2.78	** 0.006**
Parental education	0.13	0.04–0.22	2.79	**0.005**
Non-social screentime (*z*-score)	0.33	0.13–0.54	3.18	**0.001**
Childhood adversity exposome	1.10	0.87–1.34	9.16	**<0.001**
**Digital exposomic risk scores (*z*-score)**	1.78	1.58–1.98	17.41	**<0.001**
Within-group variance	20.16			
Between-group variance	family:site = 5.75, site = 0.11			
Intra-class correlation	0.23			
Marginal *R*^2^	15.61%			

*Note*. Linear-mixed-effects Model with Brief Problem Monitor (BPM) total problems *T*-scores as the dependent variable in the Testing ABCD subsample. List-wise deletion was employed for missing data. Models examined data from *n* = 4004 participants. The model included random intercepts for family (*n* = 3392) nested within 21 sites. Nakagawa’s marginal *R*^2^ indicates the variance explained by fixed effects.

Bold values are all meeting significance threshold of *P* < 0.05.

## Data Availability

This study used publicly available data from the Adolescent Brain Cognitive Development (ABCD) Study. Information on how to access ABCD data through the NIMH Data Archive (NDA) is available at https://nda.nih.gov/abcd. Code for all analyses can be found at https://github.com/barzilab1/ABCD_digital_exposome.
