# Supplementary material for: Probing the digital exposome: associations of social media use patterns with youth mental health
Source: NPP Digit Psychiatry Neurosci. 2024 Apr 23;2:5. doi: 10.1038/s44277-024-00006-9 (PMC11504934; doi:10.1038/s44277-024-00006-9)
Supplement: Supplementary file 1 — SUPPLEMENTAL MATERIAL [file 44277_2024_6_MOESM1_ESM.docx]

**Supplemental Materials**

**Probing the digital exposome:**

**associations of social media use patterns with youth mental health**

**Contents:**

**Supplementary methods**

**Table S1:** Variable list

**Table S2:** Validation models testing the association of digital exposomic risk with mental health burden in the independent testing ABCD subsample

**Table S3:** Association of the digital exposomic score with suicide attempts in the independent testing ABCD subsample

**Table S4:** Sensitivity analysis of validation models while retaining outlier values

**Table S5:** Sensitivity analysis of validation models while excluding youth who do not have a smartphone

**Table S6:** Sensitivity analysis of validation models while excluding youth who do not engage in social media

**Table S7:** Sensitivity analysis of validation models using BPM internalizing T-scores as the dependent variable (and not BPM-total score as in main analysis)

**Table S8:** Sensitivity analysis of validation models using parent-report psychopathology (Total CBCL score) as the dependent variable (and not youth self-report as in main analysis).

**Figure S1:** Correlation among social media variables

**Figure S2:** Variance Explained in Mental Health Symptoms

**Supplementary Methods**

**ABCD Exclusions**. Note that the overall ABCD Study exclusion criteria included lack of English fluency, major medical or neurological conditions, premature birth, magnetic resonance imaging (MRI) contraindication, history of traumatic brain injury, current diagnosis of schizophrenia, moderate/severe autism spectrum disorder, intellectual disability, and alcohol/substance use disorder.

**Digital Exposures**. ABCD collects youth- and parent-report data on various aspects of digital experiences. A total of 250 variables was examined and screened by the co-authors for relevance to understanding associations with social media experiences. Variables regarding non-social digital device use (e.g., for reading or schoolwork) were removed. Redundant items or those used for branching logic were removed (e.g., cybb_phenx_harm_12mo: if participants endorsed experiencing cyberbullying, they were asked, “*Has this happened to you in the past 12 months?”.* This variable was thus colinear/contingent on the main cybb_phenx_harm item and was removed from analysis). Seven social media variables with an endorsement rate of <1% were removed (**Table S1**).

Each screentime metric was assessed in ABCD with report of hours and minutes, which were combined for analysis. An example items is below regarding weekday social media use which was originally assessed via two responses but combined into one variable for analysis.

For example: ABCD Youth Screen Time Survey (abcd_stq01) – “*On a typical weekday: Visit social media apps (e.g., Snapchat, Facebook, Twitter, Instagram, TikTok, etc.? (Do not include time spent editing photos or videos to post on social media.)*”

screentime_6_wkdy_hr: response options for 0–23 hours

screentime_6_wkdy_min: response options for 0=0; 15=15; 30=30; 45=45 minutes

**Non-Social Screen Time**. A summary of typical non-social screentime (sum minutes per day) was calculated based on screentime questionnaire responses (child-report or parent-report [*_p*]) regarding weekdays (wkdy) or weekends (wknd). This included 13 items (each with 2 response fields for hours (hr) and minutes (min) that were collapsed together for analysis, the same as with the above digital exposures):

*screentime_1_wkdy_[hr+min], screentime_1_wkdy_[hr+min]_p ,screentime_1_wknd_[hr+min]_p*

*screentime_15_wknd_[hr+min], screentime_2_wkdy_[hr+min], screentime_3_wkdy_[hr+min]*

*screentime_7_wknd_[hr+min], screentime_8_wknd_[hr+min] ,screentime_9_wkdy_[hr+min]*

*screentime_9_wknd_[hr+min], screentime_wkdy_school_[hr+min], screentime_wkdy_typical_[hr+min], screentime_wknd_typical_[hr+min]*

**Outlier Removal**. Several continuous variables exhibited outlier responses that could influence results. For the main analyses, outliers were removed. Sensitivity analyses were run to confirm the main results while retaining these outliers. For all screentime variables, outliers greater than the 99^th^ percentile of each variable were removed (**Table S1**). For two variables assessing follower counts on the ABCD Youth Screen Time Survey (abcd_stq01), responses >5,000 were removed as outliers (**Table S1**).

screentime_smq_followers: *On (screentime_smq_use_most), how many followers do you have?*

screentime_smq_following: *On (screentime_smq_use_most), how many people or groups are you following?*

**Model Covariates**. All linear mixed effects models included random intercepts for family (rel_family_id; to account for siblings) nested within study site (site_id_l; 21 study locations). All models included fixed effect covariates for age (abcd_lpds01:interview_age), sex (pdem02:sex), race (pdem02:demo_race_a_p), Hispanic ethnicity (pdem02: demo_ethn_v2), household income (abcd_lpds01:demo_comb_income_v2_l), and parent education (mean of abcd_lpds01:demo_prnt_ed_v2_l and abcd_lpds01:demo_prtnr_ed_v2_l). Race/ethnicity was determined from baseline baseline; SGM was determined from 2-year follow-up data.

**Sensitivity Analyses**. The main analyses were re-run in subsets of children excluding those (a) who do not have a personal smartphone (abcd_stq01: screentime_phone_yn) and (b) do not use social media (abcd_stq01: screentime_usesoc).

**Table S1: Variable list**

| # | **Measure** | **Variable Name** | **Variable Text** | **Scoring** | **Notes** | | **Missing**  **N (%)^b^** |  |  |  |
| --- | --- | --- | --- | --- | --- | --- | --- | --- | --- | --- |
| 1 | abcd_stq01 | **screentime_4_wkdy *** | On a typical weekday: Play multiplayer video games on a computer, console, phone, or other device (Xbox, Play Station, iPad, AppleTV) where you can interact with others in the game? | 0–24 hours | outliers >99% removed (>7.75hr) | | 45 (0.89%) | **Screentime** |  |  |
| 2 |  | **screentime_5_wkdy *** | On a typical weekday: Text on a cell phone, tablet, computer, iPod, or other electronic device (e.g., GChat, Whatsapp, Kik, etc.)? | 0–24 hours | outliers >99% removed (>7hrs) | | 43 (0.85%) |  |  |  |
| 3 |  | **screentime_8_wkdy *** | On a typical weekday: Video chat (Skype, FaceTime, VRchat, etc.) | 0–24 hours | outliers >99% removed (>5hrs) | | 47 (0.92%) |  |  |  |
| 4 |  | **screentime_10_wknd *** | On a typical weekend day: Play multiplayer video games on a computer, console, phone, or other device (Xbox, Play Station, iPad, AppleTV) where you can interact with others in the game? | 0–24 hours | outliers >99% removed (>12hrs) | | 37 (0.73%) |  |  |  |
| 5 |  | **screentime_11_wknd *** | On a typical weekend day: Text on a cell phone, tablet, computer, iPod, or other electronic device (e.g., GChat, Whatsapp, Kik etc.)? | 0–24 hours | outliers >99% removed (>8hrs) | | 46 (0.91%) |  |  |  |
| 6 |  | **screentime_12_wknd *** | On a typical weekend day: Visit social media apps (e.g., Snapchat, Facebook, Twitter, Instagram, TikTok, etc.? (Do not include time spent editing photos or videos to post on social media.) | 0–24 hours | outliers >99% removed (>9hrs) | | 39 (0.77%) |  |  |  |
| 7 | abcd_stq01 | **screentime_14_wknd *** | On a typical weekend day: Video chat (Skype, FaceTime, VRchat, etc.) | 0–24 hours | outliers >99% removed (>7hrs) | | 44 (0.87%) |  |  |  |
| 8 |  | **screentime_smq_soc_med *** | How much TIME per day do you spend on social media apps? | 0–24 hours | outliers >99% removed (>7hrs) | | 33 (0.65%) |  |  |  |
| 9 |  | **screentime_smq_secret *** | Do you have a social media account that you keep secret from your parents? | 1=Yes; 0=No |  | | 2,360 (46.44%) | **Parent** |  |  |
| 10 | screentime_psq_p01 | screentime_following_child_p | Are you following or friends with your child on any social media sites (e.g. Facebook, Instagram, Snapchat)? | 1 = Yes; 0=No |  | | 125 (2.46%) |  |  |  |
| 11 |  | **screentime_secs_media_p *** | Do you suspect that your child has social media accounts that you are unaware of? | 1 = Yes; 0=No |  | | 57 (1.12%) |  |  |  |
| 12 | abcd_stq01 | **screentime_smq_facebook *** | List the number of accounts on: Facebook | 0–10 |  | | 2,346 (46.16%) | **App Type** |  |  |
| 13 |  | **screentime_smq_instagram *** | List the number of accounts on: Instagram | 0–10 |  | | 2,346 (46.16%) |  |  |  |
| 14 |  | **screentime_smq_snapchat *** | List the number of accounts on: Snapchat | 0–10 |  | | 2,346 (46.16%) |  |  |  |
| 15 |  | **screentime_smq_twitter *** | List the number of accounts on: Twitter | 0–10 |  | | 2,346 (46.16%) |  |  |  |
| 16 |  | **screentime_smq_youtube *** | List the number of accounts on: YouTube | 0–10 |  | | 2,346 (46.16%) |  |  |  |
| 17 |  | screentime_smq_pinterest | List the number of accounts on: Pinterest | 0–10 |  | | 2,346 (46.16%) |  |  |  |
| 18 |  | **screentime_smq_tumblr *** | List the number of accounts on: Tumblr | 0–10 |  | | 2,346 (46.16%) |  |  |  |
| 19 |  | **screentime_smq_reddit *** | List the number of accounts on: Reddit | 0–10 |  | | 2,346 (46.16%) |  |  |  |
| 20 |  | **screentime_smq_mg_chat *** | List the number of accounts on: Multiplayer Videogame Online Chatting | 0–10 |  | | 2,346 (46.16%) |  |  |  |
| 21 |  | **screentime_smq_musical_ly *** | List the number of accounts on: TikTok | 0–10 |  | | 2,346 (46.16%) |  |  |  |
| 22 |  | **screentime_smq_other *** | List the number of accounts on: Other | 0–10 |  | | 2,346 (46.16%) |  |  |  |
| 23 |  | screentime_smq_use_most_Instagram | Which social media site do you use the most? Instagram | 1=Yes; 0=No |  | | 2,347 (46.18%) |  |  |  |
| 24 |  | screentime_smq_use_most_Snapchat | Which social media site do you use the most? Snapchat | 1=Yes; 0=No |  | | 2,347 (46.18%) |  |  |  |
| 25 |  | screentime_smq_use_most_YouTube | Which social media site do you use the most? YouTube | 1=Yes; 0=No |  | | 2,347 (46.18%) |  |  |  |
| 26 |  | screentime_smq_use_most_Multiplayer | Which social media site do you use the most? Multiplayer Videogame Online Chatting | 1=Yes; 0=No |  | | 2,347 (46.18%) |  |  |  |
| 27 |  | **screentime_smq_use_most_TikTok *** | Which social media site do you use the most? TikTok | 1=Yes; 0=No |  | | 2,347 (46.18%) |  |  |  |
| 28 | abcd_stq01 | **screentime_smqa1 *** | I spend a lot of time thinking about social media apps or planning my use of social media apps. | 1=Never; 2=Very rarely; 3=Rarely; 4=Sometimes; 5=Often; 6=Very often |  | | 2,351 (46.26%) | **Overuse** |  |  |
| 29 |  | **screentime_smqa2 *** | I feel the need to use social media apps more and more. |  |  | | 2,348 (46.20%) |  |  |  |
| 30 |  | **screentime_smqa3 *** | I use social media apps so I can forget about my problems. |  |  | | 2,350 (46.24%) |  |  |  |
| 31 |  | **screentime_smqa4 *** | I've tried to use my social media apps less but I can't. |  |  | | 2,356 (46.36%) |  |  |  |
| 32 |  | **screentime_smqa5 *** | I've become stressed or upset if I am not allowed to use my social media apps. |  |  | | 2,352 (46.28%) |  |  |  |
| 33 |  | **screentime_smqa6 *** | I use social media apps so much that it has had a bad effect on my schoolwork or job |  |  | | 2,351 (46.26%) |  |  |  |
| 34 |  | **screentime_sq7 *** | Spend time online on social media (e.g. Facebook) | 1=0 nights in the past week; 2=1-2 nights; 3=3-4 nights; 4=5-7 nights |  | | 22 (0.43%) |  |  |  |
| 35 |  | **screentime_sq8 *** | Spend time in chat rooms |  |  | | 20 (0.39%) |  |  |  |
| 36 |  | **screentime_sq13 *** | In the past week, when you woke up during the night, how often have you used your phone or other device to send messages/play games/search or browse the internet/use social media/read or write emails? |  |  | | 1,727 (33.98%) |  |  |  |
| 37 | abcd_cb01 | **cybb_phenx_harm *** | Have you ever been cyberbullied, where someone was trying on purpose to harm you or be mean to you online, in texts, or group texts, or on social media (like Instagram or Snapchat)? | 1=Yes; 0=No |  | | 21 (0.41%) | **Peer** |  |  |
| 38 | abcd_stq01 | **screentime_smq_account *** | On (screentime_smq_use_most),  is your account public or private? | 1=Public; 0=Private | Reverse coded for analysis | | 2,713 (53.38%) |  |  |  |
| 39 | abcd_stq01 | **screentime_phone3 *** | I feel connected to others when I am using my phone | 1=Strongly Disagree – 7=Strongly Agree |  | | 1,505 (29.61%) |  |  |  |
| 40 |  | **screentime_smq_followers *** | On (screentime_smq_use_most), how many followers do you have? |  | outliers >5,000 removed | | 2,835 (55.79%) |  |  |  |
| 41 |  | **screentime_smq_following *** | On (screentime_smq_use_most), how many people or groups are you following? |  | outliers >5,000 removed | | 2847 (56.02%) |  |  |  |
| **Excluded Variables** | | |  |  |  |  | | | | |
| 42 | *abcd_cb01* | *cybb_phenx_harm2* | *Have you ever cyberbullied someone, where you purposefully tried to harm another person or be mean to them online, in texts or group texts, or on social media (like Instagram or Snapchat)?* | *1=Yes; 0=No* | **Variable removed for <1% endorsement** | | | | |  |
| 43 | *abcd_stq01* | *screentime_odq1* | *Have you ever used a dating app?* | *1=Yes; 0=No* | **Variable removed for <1% endorsement** | | | | |  |
| 44 |  | *screentime_smq_use_most_Facebook* | *Which social media site do you use the most? Facebook* | *1=Yes; 0=No* | **Variable removed for <1% endorsement** | | | | |  |
| 45 |  | *screentime_smq_use_most_Twitter* | *Which social media site do you use the most? Twitter* | *1=Yes; 0=No* | **Variable removed for <1% endorsement** | | | | |  |
| 46 |  | *screentime_smq_use_most_Pinterest* | *Which social media site do you use the most? Pinterest* | *1=Yes; 0=No* | **Variable removed for <1% endorsement** | | | | |  |
| 47 |  | *screentime_smq_use_most_Tumblr* | *Which social media site do you use the most? Tumblr* | *1=Yes; 0=No* | **Variable removed for <1% endorsement** | | | | |  |
| 48 |  | *screentime_smq_use_most_Reddit* | *Which social media site do you use the most? Reddit* | *1=Yes; 0=No* | **Variable removed for <1% endorsement** | | | | |  |
| 49 |  | *screentime_6_wkdy* | *On a typical weekday: Visit social media apps (e.g., Snapchat, Facebook, Twitter, Instagram, TikTok, etc.? (Do not include time spent editing photos or videos to post on social media.)* | *0–24 hours* | **outliers >99% removed (>7hrs); Variable removed for r>.9 colinearity** | | | | |  |
| 50 |  | *screentime_7_wkdy* | *On a typical weekday: Edit photos or videos to post on social media.* | *0–24 hours* | **outliers >99% removed (>2.2175hrs); Variable removed for r>.9 colinearity** | | | | |  |
| 51 |  | *screentime_13_wknd* | *On a typical weekend day: Edit photos or videos to post on social media.* | *0–24 hours* | **outliers >99% removed (>3hrs); Variable removed for r>.9 colinearity** | | | | |  |
| 52 |  | *screentime_usesoc* | *Do you have a at least one social media account?* | *1=Yes; 0=No* | **Variable removed for r>.9 colinearity** | | | | |  |

*Note*. 52 social media variables were screened for the current analyses; 11 were excluded for low endorsement or high collinearity (bottom panel).

* Bolded variables were significantly associated with psychopathology at the FDR-adjusted p<.05 threshold in ExWAS results.

**^a^** Missing data in the discovery sample before data imputation.

**Table S2: Validation models testing the association of digital exposomic risk with mental health burden in the independent testing ABCD subsample**

|  | **Model 1 (N = 4,012)** | | | **Model 2 (N = 4,012)** | | | **Model 3 (N = 4,004)** | | | **Model 4 (N = 4,004)^b^** | | |
| --- | --- | --- | --- | --- | --- | --- | --- | --- | --- | --- | --- | --- |
| Predictors | ***b*** | ***CI*** | ***t^a^*** | ***b*** | ***CI*** | ***t^a^*** | ***b*** | ***CI*** | ***t^a^*** | ***b*** | ***CI*** | ***t^a^*** |
| Intercept | 50.09 | 46.67 – 53.50 | 28.75*** | 51.42 | 48.10 – 54.74 | 30.35*** | 47.43 | 44.06 – 50.80 | 27.6*** | 50.52 | 47.25 – 53.79 | 30.28*** |
| Age (years) | 0.04 | 0.02 – 0.06 | 3.55*** | 0.02 | -0.00 – 0.04 | 1.51 | 0.02 | 0.00 – 0.04 | 2.17* | 0.00 | -0.02 – 0.02 | -0.17 |
| Sex (male>female) | 0.27 | -0.08 – 0.61 | 1.53 | 0.58 | 0.25 – 0.92 | 3.38*** | 0.55 | 0.22 – 0.88 | 3.22*** | 0.30 | -0.03 – 0.62 | 1.81 |
| Race-Black | -0.07 | -0.69 – 0.55 | -0.21 | -0.71 | -1.31 – -0.10 | -2.29* | -1.36 | -1.98 – -0.75 | -4.36*** | -1.46 | -2.05 – -0.87 | -4.84*** |
| Race-White | 0.15 | -0.42 – 0.71 | 0.51 | 0.21 | -0.33 – 0.76 | 0.76 | 0.32 | -0.23 – 0.86 | 1.15 | 0.39 | -0.14 – 0.91 | 1.45 |
| Hispanic ethnicity | 0.46 | -0.05 – 0.97 | 1.77 | 0.19 | -0.30 – 0.68 | 0.77 | 0.22 | -0.28 – 0.71 | 0.87 | 0.20 | -0.28 – 0.68 | 0.82 |
| Household Income | -0.12 | -0.23 – -0.02 | -2.3* | -0.06 | -0.16 – 0.04 | -1.18 | 0.13 | 0.02 – 0.23 | 2.30* | 0.15 | 0.04 – 0.25 | 2.78** |
| Parental Education | -0.09 | -0.18 – 0.01 | -1.82 | 0.00 | -0.09 – 0.09 | 0.00 | 0.10 | 0.01 – 0.20 | 2.16* | 0.13 | 0.04 – 0.22 | 2.79** |
| Non-Social Screentime (z-score) |  |  |  | 1.38 | 1.19 – 1.56 | 14.46*** | 1.18 | 0.99 – 1.36 | 12.22*** | 0.33 | 0.13 – 0.54 | 3.18*** |
| Childhood Adversity Exposome (z-score) |  |  |  |  |  |  | 1.31 | 1.07 – 1.56 | 10.65*** | 1.10 | 0.87 – 1.34 | 9.16*** |
| Digital Exposomic Risk Scores (z-score) |  |  |  |  |  |  |  |  |  | 1.78 | 1.58 – 1.98 | 17.41*** |
| Within-Group Variance (σ2) | 22.89 | | | 22.62 | | | 21.97 | | | 20.16 | | |
| Between-Group Variance (τ00) | family:site=7.32, site=0.07 | | | family:site=6.07, site=0.03 | | | family:site=5.89, site=0.09 | | | family:site=5.75, site=0.11 | | |
| Intra-Class Correlation | 0.24 | | | 0.21 | | | 0.21 | | | 0.23 | | |
| Nakagawa’s marginal R^2^ | 1.14% | | | 6.18% | | | 9.07% | | | 15.61% | | |

*Note*. Linear mixed-effects model with Brief Problem Monitor (BPM) total problems T-scores as the dependent variable in the Testing ABCD subsample. Listwise deletion was employed for missing data. Models examined data from n=4,004 participants. The model included random intercepts for family (n=3,392) nested within 21 sites. Nakagawa’s marginal R^2^ indicates the variance explained by fixed effects.

***^a^*** (*) p<0.05; (**) p<0.01; (***) p<0.001

**^b^** Model 4 is shown in the main paper in Table 2.

**Table S3: Association of the digital exposomic score with suicide attempts in the independent testing ABCD subsample**

|  | **Model 1 (N = 4,460)** | | | **Model 2 (N = 4,460)** | | | **Model 3 (N = 4,460)** | | | **Model 4 (N = 4,452)** | | | **Model 5 (N = 4,460)** | | | **Model 6 (N = 4,452)** | | |
| --- | --- | --- | --- | --- | --- | --- | --- | --- | --- | --- | --- | --- | --- | --- | --- | --- | --- | --- |
| Predictors | ***OR*** | ***CI*** | ***z^a^*** | ***OR*** | ***CI*** | ***z^a^*** | ***OR*** | ***CI*** | ***z^a^*** | ***OR*** | ***CI*** | ***z^a^*** | ***OR*** | ***CI*** | ***z^a^*** | ***OR*** | ***CI*** | ***z^a^*** |
| Age (years) | 1.05 | 1.01 – 1.08 | 2.67** | 1.04 | 1.00 – 1.07 | 2.10* | 1.02 | 0.99 – 1.06 | 1.37 | 1.04 | 1.01 – 1.08 | 2.41* | 1.02 | 0.99 – 1.06 | 1.36 | 1.03 | 1.00 – 1.07 | 1.70 |
| Sex (male>female) | 1.78 | 1.03 – 3.08 | 2.07* | 2.01 | 1.16 – 3.50 | 2.48* | 1.5 | 0.85 – 2.61 | 1.41 | 1.87 | 1.07 – 3.26 | 2.21* | 1.57 | 0.89 – 2.75 | 1.56 | 1.53 | 0.87 – 2.70 | 1.46 |
| Race-Black | 0.70 | 0.29 – 1.67 | -0.81 | 0.51 | 0.21 – 1.25 | -1.48 | 0.45 | 0.18 – 1.16 | -1.64 | 0.27 | 0.11 – 0.69 | -2.73** | 0.42 | 0.16 – 1.10 | -1.77 | 0.25 | 0.09 – 0.66 | -2.78** |
| Race-White | 0.54 | 0.25 – 1.17 | -1.56 | 0.54 | 0.25 – 1.18 | -1.53 | 0.49 | 0.22 – 1.09 | -1.75 | 0.57 | 0.26 – 1.26 | -1.39 | 0.48 | 0.21 – 1.07 | -1.80 | 0.51 | 0.23 – 1.16 | -1.6 |
| Hispanic ethnicity | 0.94 | 0.44 – 1.98 | -0.17 | 0.83 | 0.40 – 1.74 | -0.48 | 0.88 | 0.42 – 1.86 | -0.33 | 0.88 | 0.43 – 1.83 | -0.33 | 0.84 | 0.40 – 1.78 | -0.46 | 0.9 | 0.43 – 1.88 | -0.28 |
| Household Income | 0.89 | 0.77 – 1.02 | -1.63 | 0.93 | 0.80 – 1.07 | -1.07 | 0.95 | 0.82 – 1.10 | -0.70 | 1.06 | 0.91 – 1.23 | 0.69 | 0.95 | 0.83 – 1.10 | -0.63 | 1.07 | 0.91 – 1.25 | 0.80 |
| Parental Education | 0.97 | 0.85 – 1.11 | -0.40 | 0.99 | 0.87 – 1.13 | -0.14 | 1.02 | 0.88 – 1.17 | 0.23 | 1.09 | 0.94 – 1.25 | 1.13 | 1.02 | 0.89 – 1.17 | 0.25 | 1.10 | 0.95 – 1.27 | 1.25 |
| Non-Social Screentime (z-score) |  |  |  | 1.74 | 1.37 – 2.20 | 4.60*** |  |  |  | 1.48 | 1.16 – 1.88 | 3.12** | 1.21 | 0.91 – 1.62 | 1.33 | 1.10 | 0.82 – 1.47 | 0.63 |
| Digital Exposomic Risk Scores (z-score)^b^ |  |  |  |  |  |  | 2.12 | 1.74 – 2.59 | 7.42*** |  |  |  | 1.96 | 1.55 – 2.47 | 5.70*** | 1.76 | 1.39 – 2.23 | 4.69*** |
| Childhood Adversity Exposome (z-score) |  |  |  |  |  |  |  |  |  | 3.20 | 2.19 – 4.66 | 6.05 |  |  |  | 2.76 | 1.88 – 4.05 | 5.18*** |
| Within-Group Variance (σ2) | 3.29 | | | 3.29 | | | 3.29 | | | 3.29 | | | 3.29 | | | 3.29 | | |
| Between-Group Variance (τ00) | family:site= 0.90, site=0 | | | family:site= 0.87, site=0 | | | family:site= 0.54, site=0 | | | family:site= 0.44, site=0 | | | family:site= 0.56, site=0 | | | family:site= 0.32, site=0 | | |
| Intra-Class Correlation | - | | | - | | | - | | | - | | | - | | | - | | |
| Nakagawa’s R^2^ | 9.76% | | | 15.47% | | | 18.67% | | | 29.17% | | | 19.79% | | | 29.29% | | |

*Note.* All models were mixed effects logistic regression (*lme4::glmer* with “family=binomial”) and included random intercepts for family and site (3,712 families, 21 sites). Nakagawa’s marginal R^2^ indicates the variance explained by fixed effects.

***^a^*** (*) p<0.05; (**) p<0.01; (***) p<0.001

^b^ Digital Exposomic Risk Score were calculated from the ExWAS of BPM total T-scores as the outcome in the testing subsample.

**Table S4: Sensitivity analysis of validation models while retaining outlier values**

|  | **Model 1 (N = 4,012)** | | | **Model 2 (N = 4,012)** | | | **Model 3 (N = 4,004)** | | | **Model 4 (N = 4,004)** | | |
| --- | --- | --- | --- | --- | --- | --- | --- | --- | --- | --- | --- | --- |
| Predictors | ***b*** | ***CI*** | ***t^a^*** | ***b*** | ***CI*** | ***t^a^*** | ***b*** | ***CI*** | ***t^a^*** | ***b*** | ***CI*** | ***t^a^*** |
| Intercept | 50.09 | 46.67 – 53.50 | 28.75*** | 50.96 | 47.64 – 54.28 | 30.09*** | 46.95 | 43.58 – 50.32 | 27.32*** | 49.99 | 46.73 – 53.25 | 30.06*** |
| Age (years) | 0.04 | 0.02 – 0.06 | 3.55*** | 0.02 | -0.00 – 0.04 | 1.81 | 0.03 | 0.01 – 0.05 | 2.45* | 0.00 | -0.02 – 0.02 | 0.22 |
| Sex (male>female) | 0.27 | -0.08 – 0.61 | 1.53 | 0.53 | 0.19 – 0.87 | 3.05** | 0.50 | 0.17 – 0.84 | 2.94** | 0.32 | 0.00 – 0.65 | 1.97* |
| Race-Black | -0.07 | -0.69 – 0.55 | -0.21 | -0.66 | -1.26 – -0.05 | -2.13* | -1.33 | -1.95 – -0.72 | -4.26*** | -1.41 | -2.00 – -0.81 | -4.66*** |
| Race-White | 0.15 | -0.42 – 0.71 | 0.51 | 0.25 | -0.30 – 0.79 | 0.88 | 0.35 | -0.19 – 0.90 | 1.27 | 0.39 | -0.14 – 0.91 | 1.45 |
| Hispanic ethnicity | 0.46 | -0.05 – 0.97 | 1.77 | 0.3 | -0.19 – 0.78 | 1.19 | 0.3 | -0.19 – 0.80 | 1.20 | 0.25 | -0.23 – 0.73 | 1.01 |
| Household Income | -0.12 | -0.23 – -0.02 | -2.30* | -0.05 | -0.16 – 0.05 | -1.04 | 0.13 | 0.03 – 0.24 | 2.45* | 0.16 | 0.06 – 0.26 | 3.05** |
| Parental Education | -0.09 | -0.18 – 0.01 | -1.82 | -0.01 | -0.10 – 0.09 | -0.11 | 0.10 | 0.01 – 0.20 | 2.10* | 0.12 | 0.03 – 0.21 | 2.56* |
| Non-Social Screentime (z-score) |  |  |  | 1.36 | 1.17 – 1.55 | 14.05*** | 1.16 | 0.96 – 1.35 | 11.84*** | 0.22 | 0.01 – 0.43 | 2.02* |
| Childhood Adversity Exposome (z-score) |  |  |  |  |  |  | 1.33 | 1.09 – 1.58 | 10.79*** | 1.12 | 0.89 – 1.36 | 9.36*** |
| Digital Exposomic Risk Scores (z-score)^b^ |  |  |  |  |  |  |  |  |  | 1.85 | 1.65 – 2.05 | 17.97*** |
| Within-Group Variance (σ2) | 22.89 | | | 22.59 | | | 21.94 | | | 20.16 | | |
| Between-Group Variance (τ00) | family:site=7.32, site=0.07 | | | family:site=6.20, site=0.02 | | | family:site=5.98, site=0.11 | | | family:site=5.68, site=0.11 | | |
| Intra-Class Correlation | 0.24 | | | 0.22 | | | 0.22 | | | 0.22 | | |
| Nakagawa’s R^2^ | 1.14% | | | 5.89% | | | 8.89% | | | 15.79% | | |

*Note*. Linear mixed-effects model with Brief Problem Monitor (BPM) total problems T-scores as the dependent variable in the Testing ABCD subsample. Listwise deletion was employed for missing data. Models examined data from n=4,004 participants. The model included random intercepts for family (n=3,392) nested within 21 sites. Nakagawa’s marginal R^2^ indicates the variance explained by fixed effects.

Outlier values noted in Table S1 were removed for analyses in Table S2 but retained in these analyses.

***^a^*** (*) p<0.05; (**) p<0.01; (***) p<0.001

^b^ Digital Exposomic Risk Score were calculated from the ExWAS of BPM total T-scores as the outcome in the testing subsample.

**Table S5: Sensitivity analysis of validation models while excluding youth who do not have a smartphone**

|  | **Model 1 (N = 2,861)** | | | **Model 2 (N = 2,861)** | | | **Model 3 (N = 2,857)** | | | **Model 4 (N = 2,857)** | | |
| --- | --- | --- | --- | --- | --- | --- | --- | --- | --- | --- | --- | --- |
| Predictors | ***b*** | ***CI*** | ***t^a^*** | ***b*** | ***CI*** | ***t^a^*** | ***b*** | ***CI*** | ***t^a^*** | ***b*** | ***CI*** | ***t^a^*** |
| Intercept | 50.5 | 46.40 – 54.61 | 24.13*** | 51.96 | 47.98 – 55.94 | 25.6*** | 48.12 | 44.09 – 52.16 | 23.39*** | 51.11 | 47.21 – 55.02 | 25.66*** |
| Age (years) | 0.04 | 0.01 – 0.06 | 2.67** | 0.01 | -0.01 – 0.04 | 1.01 | 0.02 | -0.01 – 0.05 | 1.56 | 0.00 | -0.03 – 0.02 | -0.26 |
| Sex (male>female) | 0.4 | -0.02 – 0.82 | 1.88 | 0.76 | 0.35 – 1.17 | 3.65*** | 0.74 | 0.34 – 1.14 | 3.59*** | 0.45 | 0.06 – 0.84 | 2.26* |
| Race-Black | -0.02 | -0.74 – 0.70 | -0.06 | -0.69 | -1.40 – 0.02 | -1.92 | -1.28 | -1.99 – -0.57 | -3.52*** | -1.38 | -2.07 – -0.70 | -3.96*** |
| Race-White | 0.1 | -0.56 – 0.77 | 0.31 | 0.23 | -0.42 – 0.87 | 0.68 | 0.34 | -0.30 – 0.98 | 1.04 | 0.31 | -0.31 – 0.93 | 0.99 |
| Hispanic ethnicity | 0.39 | -0.20 – 0.98 | 1.29 | 0.13 | -0.44 – 0.70 | 0.45 | 0.18 | -0.39 – 0.75 | 0.63 | 0.13 | -0.42 – 0.69 | 0.47 |
| Household Income | -0.18 | -0.30 – -0.06 | -2.83** | -0.10 | -0.22 – 0.02 | -1.60 | 0.07 | -0.05 – 0.20 | 1.12 | 0.11 | -0.01 – 0.23 | 1.78 |
| Parental Education | -0.06 | -0.17 – 0.05 | -1.00 | 0.01 | -0.10 – 0.12 | 0.23 | 0.11 | -0.00 – 0.22 | 1.94 | 0.13 | 0.02 – 0.24 | 2.41* |
| Non-Social Screentime (z-score) |  |  |  | 1.49 | 1.27 – 1.71 | 13.22*** | 1.28 | 1.05 – 1.50 | 11.21*** | 0.48 | 0.24 – 0.71 | 3.94*** |
| Childhood Adversity Exposome (z-score) |  |  |  |  |  |  | 1.23 | 0.95 – 1.52 | 8.50*** | 1.01 | 0.73 – 1.29 | 7.15*** |
| Digital Exposomic Risk Scores (z-score)^b^ |  |  |  |  |  |  |  |  |  | 1.83 | 1.60 – 2.06 | 15.68*** |
| Within-Group Variance (σ2) | 22.35 | | | 21.45 | | | 21.02 | | | 18.74 | | |
| Between-Group Variance (τ00) | family:site=8.88, site=0.03 | | | family:site=7.97, site=0.01 | | | family:site=7.63, site=0.03 | | | family:site=7.67, site=0.06 | | |
| Intra-Class Correlation | 0.29 | | | 0.27 | | | 0.27 | | | 0.29 | | |
| Nakagawa’s R^2^ | 1.22% | | | 7.00% | | | 9.44% | | | 16.70% | | |

*Note*. Linear mixed-effects model with Brief Problem Monitor (BPM) total problems T-scores as the dependent variable in the Testing ABCD subsample. Listwise deletion was employed for missing data. All models included random intercepts for family and site (2,497 families, 21 sites). Nakagawa’s marginal R^2^ indicates the variance explained by fixed effects.

These analyses parallel results in Table S2 but exclude youth who report not having a smartphone (abcd_stq01: screentime_phone_yn).

***^a^*** (*) p<0.05; (**) p<0.01; (***) p<0.001

^b^ Digital Exposomic Risk Score were calculated from the ExWAS of BPM total T-scores as the outcome in the testing subsample.

**Table S6: Sensitivity analysis of validation models while excluding youth who do not engage in social media**

|  | **Model 1 (N = 2,174)** | | | **Model 2 (N = 2,174)** | | | **Model 3 (N = 2,170)** | | | **Model 4 (N = 2,170)** | | |
| --- | --- | --- | --- | --- | --- | --- | --- | --- | --- | --- | --- | --- |
| Predictors | ***b*** | ***CI*** | ***t^a^*** | ***b*** | ***CI*** | ***t^a^*** | ***b*** | ***CI*** | ***t^a^*** | ***b*** | ***CI*** | ***t^a^*** |
| Intercept | 50.52 | 45.67 – 55.37 | 20.42*** | 52.2 | 47.48 – 56.92 | 21.68*** | 48.49 | 43.67 – 53.32 | 19.71*** | 51.76 | 47.13 – 56.40 | 21.91*** |
| Age (years) | 0.03 | -0.00 – 0.06 | 1.68 | 0.01 | -0.02 – 0.04 | 0.5 | 0.02 | -0.02 – 0.05 | 0.99 | -0.01 | -0.04 – 0.02 | -0.48 |
| Sex (male>female) | 0.24 | -0.25 – 0.74 | 0.97 | 0.64 | 0.16 – 1.13 | 2.59** | 0.62 | 0.13 – 1.10 | 2.51* | 0.31 | -0.15 – 0.77 | 1.32 |
| Race-Black | -0.79 | -1.62 – 0.04 | -1.88 | -1.48 | -2.29 – -0.66 | -3.56*** | -1.94 | -2.76 – -1.12 | -4.62*** | -1.86 | -2.65 – -1.07 | -4.63*** |
| Race-White | 0.06 | -0.73 – 0.84 | 0.15 | 0.12 | -0.64 – 0.88 | 0.32 | 0.27 | -0.49 – 1.03 | 0.69 | 0.27 | -0.46 – 0.99 | 0.72 |
| Hispanic ethnicity | 0.27 | -0.40 – 0.93 | 0.78 | 0 | -0.65 – 0.64 | 0 | 0.08 | -0.58 – 0.73 | 0.23 | 0.13 | -0.50 – 0.75 | 0.4 |
| Household Income | -0.22 | -0.36 – -0.08 | -3.07** | -0.14 | -0.27 – -0.00 | -2.01* | 0.01 | -0.13 – 0.15 | 0.13 | 0.06 | -0.08 – 0.19 | 0.83 |
| Parental Education | 0.08 | -0.05 – 0.20 | 1.16 | 0.11 | -0.02 – 0.23 | 1.72 | 0.2 | 0.07 – 0.32 | 3.05** | 0.19 | 0.07 – 0.31 | 3.03** |
| Non-Social Screentime (z-score) |  |  |  | 1.48 | 1.23 – 1.74 | 11.41*** | 1.3 | 1.04 – 1.56 | 9.83*** | 0.47 | 0.20 – 0.74 | 3.39*** |
| Childhood Adversity Exposome (z-score) |  |  |  |  |  |  | 1.09 | 0.75 – 1.43 | 6.36*** | 0.83 | 0.51 – 1.16 | 5.05*** |
| Digital Exposomic Risk Scores (z-score)^b^ |  |  |  |  |  |  |  |  |  | 1.94 | 1.68 – 2.20 | 14.54*** |
| Within-Group Variance (σ2) | 27 | | | 25.84 | | | 25.23 | | | 21.85 | | |
| Between-Group Variance (τ00) | family:site=6.61, site=0 | | | family:site=5.86, site=0 | | | family:site=5.91, site=0.04 | | | family:site=6.60, site=0.03 | | |
| Intra-Class Correlation |  | | |  | | | 0.19 | | | 0.23 | | |
| Nakagawa’s R^2^ | 1.01% | | | 7.85% | | | 8.33% | | | 16.46% | | |

*Note*. Linear mixed-effects model with Brief Problem Monitor (BPM) total problems T-scores as the dependent variable in the Testing ABCD subsample. Listwise deletion was employed for missing data. All models included random intercepts for family and site (1,909 families, 21 sites). Nakagawa’s marginal R^2^ indicates the variance explained by fixed effects.

These analyses parallel results in Table S2 but exclude youth who report not using social media (abcd_stq01: screentime_usesoc).

***^a^*** (*) p<0.05; (**) p<0.01; (***) p<0.001

^b^ Digital Exposomic Risk Score were calculated from the ExWAS of BPM total T-scores as the outcome in the testing subsample.

**Table S7: Sensitivity analysis of validation models using BPM Internalizing T-scores as the dependent variable (and not BPM Total score as in main analysis).**

|  | **Model 1 (N = 4,332)** | | | **Model 2 (N = 4,332)** | | | **Model 3 (N = 4,324)** | | | **Model 4 (N = 4,324)** | | |
| --- | --- | --- | --- | --- | --- | --- | --- | --- | --- | --- | --- | --- |
| Predictors | ***b*** | ***CI*** | ***t^a^*** | ***b*** | ***CI*** | ***t^a^*** | ***b*** | ***CI*** | ***t^a^*** | ***b*** | ***CI*** | ***t^a^*** |
| Intercept | 49.64 | 46.46 – 52.83 | 30.56*** | 50.57 | 47.45 – 53.70 | 31.76*** | 47.81 | 44.63 – 50.99 | 29.45*** | 50.14 | 47.02 – 53.26 | 31.49*** |
| Age (years) | 0.03 | 0.01 – 0.05 | 3.34*** | 0.02 | -0.00 – 0.04 | 1.68 | 0.02 | 0.00 – 0.04 | 2.14* | 0.00 | -0.02 – 0.02 | 0.23 |
| Sex (male>female) | 0.01 | -0.31 – 0.33 | 0.08 | 0.27 | -0.05 – 0.58 | 1.64 | 0.24 | -0.07 – 0.56 | 1.51 | 0.04 | -0.27 – 0.35 | 0.24 |
| Race-Black | -0.25 | -0.83 – 0.32 | -0.86 | -0.78 | -1.35 – -0.21 | -2.67** | -1.25 | -1.83 – -0.67 | -4.23*** | -1.31 | -1.88 – -0.74 | -4.52*** |
| Race-White | 0.21 | -0.32 – 0.74 | 0.79 | 0.25 | -0.26 – 0.77 | 0.97 | 0.34 | -0.18 – 0.85 | 1.28 | 0.38 | -0.12 – 0.88 | 1.49 |
| Hispanic ethnicity | 0.23 | -0.25 – 0.71 | 0.94 | 0.02 | -0.45 – 0.48 | 0.08 | 0.01 | -0.46 – 0.48 | 0.03 | 0.01 | -0.45 – 0.47 | 0.04 |
| Household Income | -0.05 | -0.15 – 0.05 | -0.98 | 0.00 | -0.10 – 0.10 | 0.01 | 0.13 | 0.03 – 0.23 | 2.53* | 0.14 | 0.04 – 0.24 | 2.82** |
| Parental Education | -0.06 | -0.15 – 0.03 | -1.31 | 0.01 | -0.08 – 0.10 | 0.22 | 0.08 | -0.01 – 0.17 | 1.84 | 0.11 | 0.02 – 0.20 | 2.46* |
| Non-Social Screentime (z-score) |  |  |  | 1.12 | 0.94 – 1.29 | 12.51*** | 0.97 | 0.79 – 1.15 | 10.69*** | 0.32 | 0.12 – 0.51 | 3.16** |
| Childhood Adversity Exposome (z-score) |  |  |  |  |  |  | 0.96 | 0.72 – 1.19 | 8.11*** | 0.77 | 0.55 – 1.00 | 6.72*** |
| Digital Exposomic Risk Scores (z-score)^b^ |  |  |  |  |  |  |  |  |  | 1.39 | 1.20 – 1.58 | 14.24*** |
| Within-Group Variance (σ2) | 19.91 | | | 19.69 | | | 19.35 | | | 18.20 | | |
| Between-Group Variance (τ00) | family:site=8.22, site=0.11 | | | family:site=7.44, site=0.06 | | | family:site=7.37, site=0.13 | | | family:site=7.37, site=0.09 | | |
| Intra-Class Correlation | 0.29 | | | 0.28 | | | 0.28 | | | 0.29 | | |
| Nakagawa’s R^2^ | 0.50% | | | 4.10% | | | 5.70% | | | 10.00% | | |

*Note*. Linear mixed-effects model with Brief Problem Monitor (BPM) Internalizing problems T-scores as the dependent variable in the Testing ABCD subsample. Listwise deletion was employed for missing data. All models included random intercepts for family and site (3,625 families, 21 sites). Nakagawa’s marginal R^2^ indicates the variance explained by fixed effects.

These analyses parallel results in Table S2 but focus on internalizing rather than total problems scores.

***^a^*** (*) p<0.05; (**) p<0.01; (***) p<0.001

^b^ Digital Exposomic Risk Score were calculated from the ExWAS of BPM total T-scores as the outcome in the testing subsample.

**Table S8: Sensitivity analysis of validation models using parent-report psychopathology (Total CBCL score) as the dependent variable (and not youth self-report as in main analysis)**

|  | **Model 1 (N = 3,513)** | | | **Model 2 (N = 3,513)** | | | **Model 3 (N = 3,513)** | | | **Model 4 (N = 3,513)** | | |
| --- | --- | --- | --- | --- | --- | --- | --- | --- | --- | --- | --- | --- |
| Predictors | ***b*** | ***CI*** | ***t^a^*** | ***b*** | ***CI*** | ***t^a^*** | ***b*** | ***CI*** | ***t^a^*** | ***b*** | ***CI*** | ***t^a^*** |
| Intercept | 33.38 | 26.33 – 40.44 | 9.27*** | 33.91 | 26.90 – 40.91 | 9.49*** | 25.99 | 18.90 – 33.08 | 7.19*** | 27.15 | 20.05 – 34.25 | 7.5*** |
| Age (years) | 0.08 | 0.04 – 0.13 | 3.76*** | 0.06 | 0.02 – 0.11 | 2.9** | 0.08 | 0.03 – 0.12 | 3.47*** | 0.07 | 0.02 – 0.11 | 2.97** |
| Sex (male>female) | -0.36 | -1.07 – 0.36 | -0.99 | -0.01 | -0.72 – 0.70 | -0.03 | -0.07 | -0.78 – 0.63 | -0.2 | -0.17 | -0.88 – 0.53 | -0.48 |
| Race-Black | 0.66 | -0.73 – 2.05 | 0.93 | 0.05 | -1.33 – 1.44 | 0.07 | -1.12 | -2.51 – 0.26 | -1.59 | -1.13 | -2.52 – 0.25 | -1.6 |
| Race-White | 3.24 | 2.00 – 4.47 | 5.14*** | 3.32 | 2.10 – 4.54 | 5.32*** | 3.7 | 2.49 – 4.91 | 6*** | 3.73 | 2.52 – 4.93 | 6.06*** |
| Hispanic ethnicity | -0.16 | -1.33 – 1.00 | -0.27 | -0.31 | -1.47 – 0.85 | -0.53 | -0.58 | -1.73 – 0.56 | -1 | -0.57 | -1.71 – 0.58 | -0.97 |
| Household Income | -0.55 | -0.78 – -0.32 | -4.67*** | -0.47 | -0.70 – -0.24 | -3.98*** | -0.12 | -0.35 – 0.12 | -0.95 | -0.11 | -0.35 – 0.12 | -0.93 |
| Parental Education | 0.08 | -0.13 – 0.28 | 0.72 | 0.17 | -0.03 – 0.38 | 1.64 | 0.4 | 0.19 – 0.61 | 3.75*** | 0.42 | 0.21 – 0.63 | 3.96*** |
| Non-Social Screentime (z-score) |  |  |  | 1.61 | 1.21 – 2.01 | 7.94*** | 1.2 | 0.80 – 1.60 | 5.88*** | 0.79 | 0.33 – 1.24 | 3.42*** |
| Childhood Adversity Exposome (z-score) |  |  |  |  |  |  | 2.72 | 2.18 – 3.26 | 9.92*** | 2.61 | 2.07 – 3.15 | 9.49*** |
| Digital Exposomic Risk Scores (z-score)^b^ |  |  |  |  |  |  |  |  |  | 0.89 | 0.45 – 1.34 | 3.93*** |
| Within-Group Variance (σ2) | 46.32 | | | 46.33 | | | 45.38 | | | 45.11 | | |
| Between-Group Variance (τ00) | family:site=69.71, site=1.53 | | | family:site=67.40, site=1.49 | | | family:site=65.11, site=1.67 | | | family:site=64.95, site=1.68 | | |
| Intra-Class Correlation | 0.61 | | | 0.60 | | | 0.60 | | | 0.60 | | |
| Nakagawa’s R^2^ | 1.79% | | | 3.50% | | | 6.91% | | | 7.27% | | |

*Note*. Linear mixed-effects model with parent-reported Child Behavior Checklist (CBCL) Total T-scores as the dependent variable in the Testing ABCD subsample. Listwise deletion was employed for missing data. All models included random intercepts for family and site (2,935 families, 21 sites). Nakagawa’s marginal R^2^ indicates the variance explained by fixed effects.

These analyses parallel results in Table S2 but focus on parent-report of total problems rather than youth self-report.

***^a^*** (*) p<0.05; (**) p<0.01; (***) p<0.001

^b^ Digital Exposomic Risk Score were calculated from the ExWAS of BPM total T-scores as the outcome in the testing subsample.

**Figure S1: Correlation among social media variables**


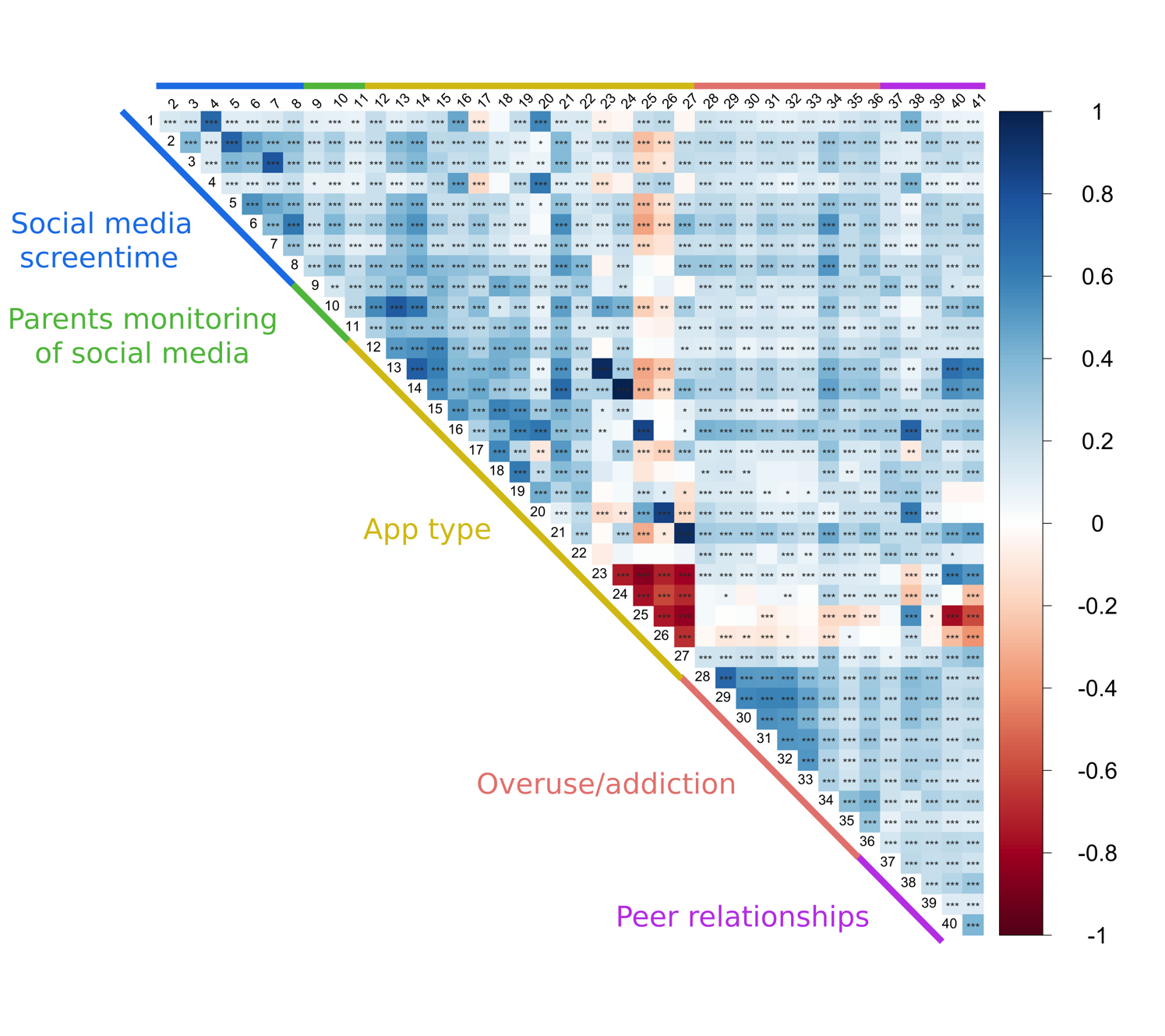


*Note*. The correlations (Pearson’s r) among the 41 social media variables used in the main analyses are shown here. The numbering of variables matches that shown in Table S1.

(*) p<0.05; (**) p<0.01; (***) p<0.001

**Figure S2: Variance Explained in Mental Health Symptoms**.


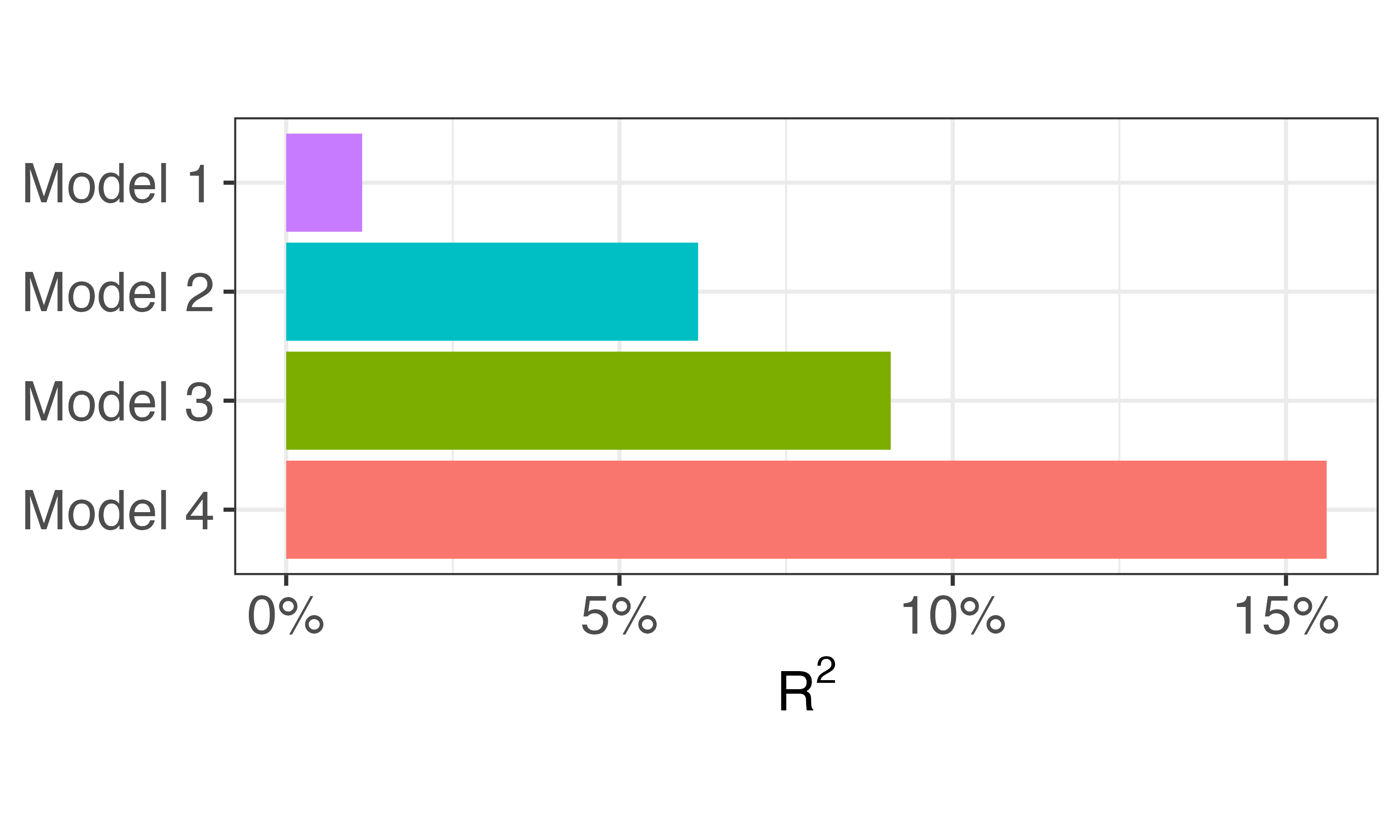


This figure displays the variance in BPM total T-scores explained by fixed effects (marginal R^2^) in the successive linear mixed-effects models in the testing subsample. Model-1 included age, sex, race, and ethnicity. Model-2 added non-social screentime. Model-3 added childhood adversity exposure. Model-4 added the digital exposomic risk scores derived from the ExWAS analyses in the training sample.
